# Reovirus Infections in Broiler Chickens: A Narrative Review

**DOI:** 10.3390/vetsci12111021

**Published:** 2025-10-22

**Authors:** George-Andrei Călugărița, Iasmina Luca, Radu-Valentin Gros, Tudor-Mihai Căsălean, Alexandru Gavrilă, Adrian Stancu

**Affiliations:** 1 Department of Anatomic Pathology and Forensic Medicine, Faculty of Veterinary Medicine, University of Life Science “King Mihai I”, Calea Aradului Street, Number 119, 300645 Timisoara, Romania; 2 Department of Microbiology, Faculty of Veterinary Medicine, University of Life Science “King Mihai I”, Calea Aradului Street, Number 119, 300645 Timisoara, Romania; valentingros@usvt.ro; 3 Department of Surgery, Faculty of Veterinary Medicine, University of Life Science “King Mihai I”, Calea Aradului Street, Number 119, 300645 Timisoara, Romania; tudorcasalean@gmail.com

**Keywords:** avian orthoreovirus, enteric viruses, malabsorption, viral identification, prophylaxis

## Abstract

**Simple Summary:**

Diseases caused by avian reoviruses remain a significant problem for the poultry industry, especially in intensively raised broilers. These viruses can affect multiple organs and functions, but the digestive tract and joints are most commonly involved, leading to considerable economic losses through mortality, growth retardation, and reduced meat quality. This paper reviews the causes, epidemiology, and clinical signs of this disease, as well as the post-hatch development of the chicken digestive system and its influence on susceptibility to enterotropic viral infections. Modern diagnostic methods are discussed, including advanced genetic techniques that enable rapid and accurate detection of circulating strains. Prevention and control strategies are also addressed, ranging from strict hygiene measures to the use of vaccines. Because these viruses frequently undergo genetic changes, existing vaccines do not always provide complete protection, and locally adapted solutions may prove more effective. Malabsorption syndrome may result from multiple concurrent enteric infections, with avian orthoreovirus playing an important but not exclusive role. The main conclusion is that these infections continue to pose a major challenge to poultry farming, and continuous monitoring, modern diagnostic tools, and the development of new vaccines are essential to safeguard bird health, ensure high-quality meat production, and support food security.

**Abstract:**

Infections caused by avian orthoreovirus represent an emerging problem with a major impact on the global poultry industry, especially in the intensive rearing of broilers. This article addresses, in a complex manner, the etiology of some clinical syndromes of interest in poultry farming: malabsorption syndrome and arthritis/tenosynovitis syndrome. Data are presented, starting from the development and physiology of the digestive tract in broiler chickens in the post-hatch period, epidemiological data, clinical signs, morphopathological changes in the intestine, and diagnostic methods in orthoreovirus infections. The development of the digestive tract is influenced by factors such as diet, digestive enzymes, intestinal pH, and intestinal microbiome/virome. Avian orthoreoviruses, belonging to the *Reoviridae* family, are double-stranded RNA viruses with multiple tropism. Phylogenetic analysis revealed the existence of at least six major genotypes, with a heterogeneous geographical distribution and genetic diversity that complicates control measures with vaccination. Characterization of the intestinal virome of broilers highlights many other enteric viruses, in addition to reoviruses, with pathogenic potential in triggering malabsorption syndrome. Thus, we can state that the etiology of malabsorption syndrome is not unitary, with the association of several viruses with intestinal tropism aggravating the clinical signs. The article describes viral identification methods, including classical techniques and advanced next-generation sequencing (NGS) approaches, used to characterize the intestinal virome and emerging pathogens. Finally, for prophylaxis, autogenous vaccines adapted to local circulating strains are recommended. Frequent genetic recombinations and high antigenic variation require continuous monitoring and constant adaptation of immunization schedules to control the disease.

## 1. Introduction

In recent decades, the poultry industry has made significant progress, especially in broiler breeding, with the aim of increasing the yield in meat production. Given the efficient feed conversion rate and good quality protein, poultry meat represents a safe source in human nutrition [1]. The improvement of these qualitative indicators in broiler chickens is largely due to genetic selection, which has determined an accelerated growth rate. However, for chickens to reach their genetic potential, it is essential that their digestive system develops properly, and this process begins immediately after hatching [2]. The physiology of broiler digestion is influenced by a number of factors, such as the development of the gastrointestinal tract; the capacity to secrete digestive enzymes; and, last but not least, the influence of the intestinal microbiome/virome [1,2,3].

Infections with avian reoviruses are contagious and of great economic importance for the poultry industry, especially intensive and extensive ones, given the variety of clinical manifestations that these pathogens induce, resulting in mortality losses, weight loss, meat depreciation, and bird welfare [4]. They mainly affect chickens, but they can also be found in turkeys, ducks, quails, geese, and wild birds (magpies, parrots, Indian spotted ducks, and other breeds of ducks and geese), and they can have from an inapparent evolution to well-evident clinical signs [4,5]. The main diseases that avian reoviruses cause are arthritis/tenosynovitis syndrome (viral arthritis) and malabsorption syndrome. However, these viruses have also been isolated from birds with chronic respiratory, neurological, enteric, hepatic, and cardiac diseases [5,6]. Even though 80% of reoviruses are considered non-pathogenic and are isolated from clinically healthy birds, the variety of hosts and clinical manifestations of these viruses denotes the insufficiency of data regarding the pathogenesis of reovirus infection [4,5].

In human medicine, reovirus was identified in 1950 in children, being the first segmented double-stranded RNA virus described. Regarding avian reoviruses, Fahey and Crawley isolated them in 1954 with the first avian reovirus from birds with chronic respiratory disease, which were structurally characterized much later, in 1976, by Spandidos and Graham [5,7].

Therefore, this review details the physiology of digestion in birds, also providing recent information from the specialized literature regarding the viral agents responsible for the two syndromes. Most of the time, the infections are mixed, with orthoreoviruses not being the only ones involved in the occurrence of this disease in broilers. Isolation and diagnostic methods are vital for poultry, and the article details all of these stages, with examples of both standard diagnostic methods and some that involve advanced research.

## 2. Physiology and Development of the Digestive Tract in Broilers in the Post-Hatching Period

After hatching, the gastrointestinal tract of the broiler is immature and requires rapid transformations for optimal digestion and absorption of nutrients from complex feeds. A growth rate of up to 300% in the first 7 days of life entails irreversible changes in the physiology of the broiler [2,8]. The gastrointestinal tube will increase its capacity given the increasing feed intake, and implicitly, the absorption surface will increase with the need for digestive enzymes and efficient nutrient transport mechanisms. It was found that the maximum growth rate in the first 5 days of life is 20%, gradually decreasing to 16% at the age of two weeks [1,2]. The rapid changes that accompany this growth rate (2.6 kg/body at 35 days of age) come with immunological and physiological immaturity, affecting the body’s ability to cope with stress and adapt to the environment, favoring the development of various infectious, metabolic, and nutritional diseases [1,2,8,9].

The residual yolk sac is the structure that provides the chick with the energy source needed in the first days after hatching, even in the absence of food. The mechanism of assimilation of nutrients from the residual yolk has still not been elucidated today; there are only hypotheses: the release of nutrients into the digestive tract or directly into the bloodstream [1,2]. The residual yolk represents between 8 and 11% of the chick’s weight at hatching and decreases to below 1% at the age of one week. In the first 3 days of life, approximately 75% of the residual yolk is exhausted [1,2]. Immediately after hatching, the chick’s digestive system develops much faster than other internal organs, regardless of whether the chicks are fed or not, but with a higher growth rate in fed chicks. This is how the transition from lipids provided by the yolk sac to carbohydrates from the feed is achieved. It has been reported that the weight of the digestive tract exceeds the body weight of the chick in the first 14 days of life. According to several authors, the maximum growth of the digestive tract occurs up to the age of 8 days, after which a decline sets in. The muscular stomach is the largest organ of the chick’s digestive tract after hatching, larger than other viscera. Organs such as the liver or pancreas seem to have a higher growth rate after 10–15 days of age, subsequently regressing [1,2].

From a microscopic point of view, the accelerated development of the intestinal mass causes changes, such as increased thickness in the intestinal submucosa, depth of the crypts, and height of the intestinal villi [2]. These changes are essential to increase the intestinal absorption surface of nutrients and vary with the intestinal segment. The rate of development of the small intestine segments differs depending on age. Thus, in the first 4 days of life, the development of the jejunum and ileum is carried out at a rate of 25%, reaching a rate of 100% at the age of 10 days. Complete growth of the duodenal intestinal villi is completed at the age of 7 days, and the other segments continue to grow up to the 10th day of life [1]. The depth of the crypts continues to increase 2–3 times with age, but the number of enterocytes does not vary. Maturation of the intestinal mucosa is achieved through cell proliferation that is directly proportional to the growth of intestinal villi and crypts. The phenomenon of cell proliferation is closely linked to the optimal feed intake, such that in batches of chickens fed improperly, the rate of cell proliferation slows down, recovering through correct feed intake [1,2]. Breeds genetically selected for high meat production yield (heavy breeds/broilers) show differences in the development of the digestive tract compared to light breeds (eggs). In broilers, both the weight and length of the digestive tract, the volume of villi, the number of enterocytes, and the enzymatic secretion/gram of feed have demonstrated higher values compared to chickens of the same age but belonging to light breeds [1,3,9].

Intestinal pH plays an essential role in the activity of digestive enzymes and in the balance of microbiota, thus influencing the digestive efficiency of broilers [1]. The variability of pH in the first weeks of life is determined by factors such as hydrochloric acid secretion, food intake, and feed composition (including the type of limestone used in the ration) [1]. Although digestive secretions increase with age, increasing the consumption of food with a neutral pH can counterbalance the acidity, leading to a progressive alkalization of the intestinal environment after day 14 of life [1,2]. Digestion and absorption of nutrients after hatching are developmental processes, influenced by both enzymatic secretion and the capacity of intestinal transport systems. Although some studies suggest that these systems are able to adequately support the nutritional needs of chicks in the first days of life, others indicate possible limitations, especially in breeds selected for rapid growth. In the first days after hatching, the capacity to digest proteins and lipids remains reduced, although the activity of some intestinal enzymes increases during incubation [1,2]. The metabolic rate of nutrients is higher when chicks receive food earlier, the optimal time for access to feed being between 24 and 30 h after hatching [8]. In the first week of life, broilers show limitations in the digestion of lipids, which they do not digest efficiently. The amount of bile salts increases up to 10-fold in the first 21 days after hatching. Pancreatic lipase takes approximately 16 days to reach a constant value, while enzymes such as amylase or trypsin reach maximum secretion at 4 days post-hatch. Therefore, in the first weeks of life, digestion and absorption of fats are deficient [1,2]. Regarding the absorption of lipids, represented mainly by triglycerides, short-chain fatty acids are absorbed directly by passive diffusion, but long chains require the binding of bile salts and the development of structures called micelles in order to be absorbed efficiently. Bile salts will be absorbed at the ileum level, and fats at the jejunum level. Both reach the liver via the portal vein, where lipids will be split with the help of lipoprotein lipase into free fatty acids and monoacylglycerol. Free fatty acids can be stored in the form of triglycerides in muscle fat [1,10]. Limited fat absorption is a phenomenon that affects chicks after hatching, and it is caused by a delay in feeding, the immaturity of the digestive tract, and the stress of transport and handling. Thus, strategies have been developed that enhance the use of fats at the intestinal level, such as supplementation with saturated or unsaturated fats, enzymes, or exogenous emulsifiers [1,2,11]. The rate of digestion (the speed at which digestion is carried out) is inversely proportional to the efficient absorption of nutrients. Maintaining a longer digestion time for contents allows for better contact with enzymes in the mucosa. Immediately after hatching, the amount of feed ingested increases, while the digestion rate decreases by up to 30% in the first 10 days of life, subsequently maintaining a plateau for the rest of the chicken’s life [2].

Digestion is a mechanism influenced by the size of the feed and the various cereals that compose the ration, as well as by reverse peristalsis. It has been found that the finer the feed is ground, the more efficiently digestion occurs. Therefore, feeding with pellets increases the performance of broilers while also decreasing their waste [12]. Motility and transit improve with the age of the chicks [2,13]. Non-starch polysaccharides (dietary fibers) represent a challenge in the digestion process in birds due to the lack of endogenous enzymes to degrade them. Thus, the stagnation of these compounds in the intestine can slow down the absorption of nutrients. The addition of new-generation carbohydrases in diets containing dietary fiber has led to better fiber digestibility, increased intestinal peristalsis, and reduced pathogenic microflora [14]. Fiber, long considered an anti-nutritional factor in the diet of broiler birds, promotes the development of the digestive tract, microbiome, and growth performance, improving nutrient absorption [15].

The maximum utilization of feed proteins is recorded when the ratio between essential amino acids and real protein has a value of 0.60. Of the total amino acids, 60% represent essential amino acids and 40% non-essential. Maintaining this amino acid/protein ratio in feed ensures the growth performance of chickens and reduces nitrogen emissions into the environment [16]. The addition of 20% microalgae (*Chlorella vulgaris*) to the feed ration leads to a decrease in the fat content of the meat and an increase in the protein content, while also changing the chemical composition of the meat. The values of some amino acids (threonine/arginine) increase, as well as the bioavailability of some minerals and vitamins (calcium, magnesium, phosphorus, and vitamin K) [17].

The protein requirement for broilers is between 180 and 230 g/kg. The degree of digestibility and absorption of these is closely related to the enteral bioavailability of the protein/amino acid and starch/glucose ratios, which favor the absorption and, ultimately, the efficiency of the feed. Thus, the use of rapidly digestible proteins (e.g., whey proteins) or starch can improve nitrogen retention, muscle proteins, and feed conversion efficiency through a balanced supply of glucose and amino acids in different segments of the intestine. Protein digestibility and absorption vary greatly in broilers depending on the chosen protein source, as well as on the analyzed intestinal segment. The degree of digestibility is a criterion based on which the quality of the feed proteins is established. Therefore, bone meal has the highest degree of digestibility in the proximal jejunum, and the ileum is the place where the highest digestibility of plasma meals has been recorded. Canola and rapeseed meals have the best digestibility rates in the distal ileum. The lowest digestibility rates have been recorded for blood meal and pressed rapeseed [18].

The use of carbohydrates, namely, oxidized glucose, as dietary supplements has led to superior growth performance compared to chicken batches where antibiotics are used as growth promoters. Also, the addition of oxidized glucose supports the integrity of the immunological, intestinal, and mucosal barriers, in contrast to diets that use antibiotics. Given that glucose favors intestinal bacterial diversity, its addition to broiler diets can replace antibiotics [19]. The inclusion of starch in broiler feed contributes to the efficient metabolism of glucose and lipids, leading to lower cholesterol (LDL) and efficient energy use during the growth phase [20,21]. Glucose absorption at the intestinal level remains a phenomenon that is not fully understood. Absorption variations are significant depending on the age of the chicks, with a decrease in intestinal glucose transport in 5-week-old chicks, even though their villi show hypertrophy, compared to 1-week-old chicks [21,22]. Under stress conditions, supplementation of drinking water with 6% glucose leads to an increase of over 3% in energy intake and improves carcass quality at slaughter [23]. Supplementation with glucose oxidase at a dose of 100 U/g increases the growth performance, antioxidant capacity, and intestinal health of broilers [24]. Even though fructose administration does not induce food depression like glucose, high doses of fructose can promote the production of large amounts of uric acid and its precipitation at the joint level [25]. The use of antimicrobials as growth promoters has not led to improved growth performance in birds [3]. Although their use reduces the density of potentially pathogenic bacteria (*Escherichia coli*), resistance to more than one class of antimicrobials has been observed in these chickens [3].

## 3. Etiology and Systematics of Avian Orthoreovirus

Currently, the International Committee on Taxonomy of Viruses systematizes the *Reoviridae* family into two subfamilies: *Sedoreoviridae* and *Spinareoviridae*. Members of these families have been isolated from mammals, birds, reptiles, crustaceans, arthropods, algae, various fungi, and plants [26,27]. Orthoreovirus avis, the species isolated from birds, belongs to the genus *Orthoreovirus* (along with nine other species), subfamily *Spinareoviridae*, family *Reoviridae*, order *Reovirales* [28]. The four strains frequently isolated from intensively raised broilers and against which vaccination is carried out are S1133, 1733, 2408, and 2177 [8,9].

### 3.1. Morphology

The avian orthoreovirus has an icosahedral particle lacking a lipid envelope, with a diameter between 75 and 85 nm, and comprises 10 double-stranded RNA segments. These segments, depending on their weight, are classified into three classes: L (large), M (medium), and S (small). The genes of each class encode specific proteins; thus, class L includes lambda proteins (λ), class M includes mu proteins (μ), and class S includes sigma proteins (σ). Each polypeptide specific to a class is referred to by an alphabetical index (λA, λB, μB, σC, etc.). The outer capsid is composed of the following proteins: μB, σB, σC, μBC, and μBN. Of these, μB represents the predominant protein and morphologically corresponds to μ 1C, identified in mammalian reovirus [29]. The internal proteins are represented by λA, μA, λB, and σA. The virion is composed of the proteins A A (145.000 Daltons), A B (130.000 Daltons), and A C (115.000 Daltons). The proteins λ A and λ B are identified on the surface of the virion. Of all the σ proteins, σC has the lowest molecular weight; it is part of the outer capsid and is the homologue of σ1 from mammals. In the case of strain S1133, several additional polypeptide bands appear, σ B, σ NS, and σ TC, with molecular weights ranging between 34.000 and 35.000 Daltons [29]. The relationship between their morphological similarities is not fully understood; even though many proteins of the avian orthoreovirus resemble those of the reoviruses isolated from mammals, there are differences that may influence the physiopathological mechanism of infection in birds [29,30,31].

### 3.2. Epidemiological Characteristics

Reoviruses have been isolated from birds with respiratory or joint diseases since 1954; later, after the 1970s, they have also been isolated from birds with malabsorption syndrome or other enteric diseases. After the 1980s, the area was expanded, and reovirus isolation was also possible from other bird species, such as Muscovy ducks, wild ducks with diarrheal syndrome, pigeons, and psittacines with liver diseases [7].

Currently, avian reoviruses continue to cause economic damage given their global distribution. The heterogeneous distribution of this infection is caused by the rapid mutations that the viral agent undergoes, as well as by the ability to infect wild birds (natural reservoirs), which significantly complicates the control of this disease [5]. Of the six distinct genotypes identified based on the σC gene coding, genotypes I and IV appear to have the greatest worldwide distribution. Genotype I is frequently isolated from birds with respiratory disease, and genotype IV is frequently isolated from arthritis/tenosynovitis lesions, while genotype VI has been identified in a wide range of lesions (arthritis/tenosynovitis to malabsorption). Current research suggests variability not only within the same genotype, but also between genotypes, with data supported by the wide range of clinical signs. Given these facts, vaccine formulations must contain all six genotypes of avian reovirus to ensure total immunization, and the effectiveness of vaccination must be supported by continuous virological and serological monitoring [5].

Although at least six major genotypes have been identified based on the σC gene, clear differences among them in terms of pathogenicity, tissue tropism, and host range have not yet been systematically established. Available data suggest a partial association between certain genotypes and clinical manifestations; however, intragenotypic variability and the lack of standardized comparative studies render these correlations inconclusive.

Viral replication, although not fully elucidated, occurs in the cytoplasm of host cells, where it causes the formation of specialized structures called viral replication organelles. These structures arise through the reorganization of cellular components, especially the endoplasmic reticulum (ER), and facilitate efficient multiplication and assembly of new viral particles. This process involves non-structural viral proteins (such as σNS and µNS) and structural proteins (such as µ2), which play a role in the formation of these compartments and in the control of replication [11]. The effect of replication is cytopathic, forming syncytia and intracytoplasmic inclusions [30].

In the case of viral arthritis and tenosynovitis, morbidity can reach up to 100% of the flock, but mortality remains low, at around 2% [30,32]. The age at which chickens are most vulnerable is 1–2 weeks, up to the age of 14–16 weeks, with the manifestations of clinical signs being pronounced. Over 94% of commercial broiler flocks are seropositive for avian reovirus based on ELISA testing, although the presence of antibodies is not always associated with clinical signs or specific lesions of the disease [33]. The horizontal transmission route is the most important, that is, through fecal/oral contact, followed by respiratory transmission or contact of viral particles with skin lesions. Eggs of sick birds represent the vertical transmission route of the infection, the reovirus having been isolated from experimentally infected embryos [7,30,34,35]. Sick birds represent the main sources of infection, with the virus remaining confined at the joint level up to 280 days post-infection. Elimination of the virus via the fecal route is achieved in 2–3 weeks, remitting for up to 7 weeks. Wild birds should not be neglected either, as they represent a natural reservoir, ensuring the dissemination of various viral genotypes in the environment and, in the absence of biosecurity rules, even poultry farms [30,36]. The incidence of infection is increased in spring/autumn and lower in winter/summer. Avian reoviruses do not have a hemagglutinating effect, unlike those of mammals. They are extremely resistant in the environment, at temperatures up to 50 °C, and are resistant to disinfectants, such as ether and chloroform, but are inactivated by ethyl alcohol (70%) and iodinated compounds [5,30].

## 4. Malabsorption Syndrome

Malabsorption syndrome is a significant pathology in broiler chicken production due to economic losses, low feed conversion rates, and low weight gain in birds. Although over the years an attempt has been made to establish an exact etiology, this has not been fully elucidated even today, which is why malabsorption syndrome is considered a multifactorial disease [1]. Many enteric viruses have been isolated from broilers with malabsorption. Of these, the genus *Orthoreovirus* represents the most frequently isolated viral genus [37,38,39,40], presenting six distinct genotypes, without an exact correlation between genotype and geographical location at a global level [5]. Other frequently isolated viral families are represented by *Sedoreoviridae* (genus *Rotavirus*), *Picornaviridae*, *Anelloviridae*, *Parvoviridae*, *Astroviridae*, *Caliciviridae, Picobirnaviridae*, *Circoviridae*, *Adenoviridae*, and *Coronaviridae* [38,41,42,43,44,45,46]. Analysis of the intestinal virome of healthy/malabsorption syndrome-affected broilers aged 2–3 weeks reveals 31 identified viral genera and 7 unclassified genera. The most frequently isolated viral families are represented by *Astroviridae*; *Caliciviridae*; *Parvoviridae*; *Picornaviridae*; *Coronaviridae*; and two families that were later abolished, *Siphoviridae* and *Myoviridae*. Currently, the latter belong to the *Caudoviricetes* class, which includes all tailed archaeal viruses. Of the total samples from chickens affected by malabsorption, the most abundant viral families identified are represented by *Picornaviridae* and *Astroviridae*. The *Parvoviridae* and *Caliciviridae* families have a higher frequency of isolation in samples collected from healthy broilers. A uniform spread has been recorded in the *Picornaviridae*, *Astroviridae*, *Coronaviridae*, and *Reoviridae* families, which have a low frequency of isolation in healthy chickens [41].

In the studies carried out to date, all the previously mentioned viral agents were isolated both from birds affected by malabsorption syndrome and from healthy birds. This shows that malabsorption syndrome does not have a unitary etiology, but rather, co-infections with enteric viruses lead to the appearance of clinical signs. The use of conventional cultivation and isolation techniques can be difficult with regard to the enteric viruses involved in malabsorption syndrome. Thus, to determine the intestinal virome as a whole, next-generation sequencing (NGS) technologies have the advantage of analyzing the entire genome, as they sequence millions of DNA/RNA fragments [16,47]. In contrast, polymerase chain reaction (PCR or RT-PCR) analyzes only specific DNA fragments, known and defined in advance, but is faster and cheaper than NGS. However, due to the unavailability of ribosomal RNA (rRNA), amplicon sequencing cannot be performed, as is applied in bacterial genomics. The use of shotgun sequencing is preferred for the identification of new viruses [16]. Bacteriological examination reveals the existence of both intestinal and extraintestinal bacterial colonies, with a high prevalence of *E. coli* bacteria, found in various viscera and even in the bone marrow of birds. At the intestinal level, bacterial species such as *Streptococcus* spp., *Lactobacillus* spp., *Pseudomonas* spp., *Enterobacter* spp., *Enterococcus* spp., and *Clostridium* spp. occupy the largest share [30,48].

### Modulation of the Avian Immune Response by Orthoreoviruses

In broilers infected with ARV, recent molecular studies indicate that the virus can directly affect the intestinal epithelium and local immune responses, disrupting the expression of genes associated with the mucosal/epithelial barrier and cytokine regulation. Experimental data suggest that ARV may induce immunosuppression by reducing T and B lymphocyte activity and altering the expression of pro- and anti-inflammatory cytokines, thereby compromising immune balance [49]. In parallel, the effects of the ARV S1133 vaccine in broilers have demonstrated intestinal histological alterations, villus shortening, and enteric atrophy, reflecting an impact on tight junction integrity and nutrient absorption, which may contribute to malabsorption syndrome [50]. Furthermore, it has been hypothesized that these disruptions may promote intestinal microbiota imbalance, potentially exacerbated by associated co-infections; however, direct evidence in broilers remains limited and warrants further targeted investigation [46,51].

## 5. Clinical Evolution of Orthoreovirus Infections

There is an extensive distribution of reoviruses in tissues (digestive, respiratory, and articular) as early as the 4th day post-infection experimentally via the respiratory/oral route. Viremia is observed even in the first 24 h after infection, the evolution of the disease being acute, chronic, or inapparent [7,30].

Clinical signs in the case of arthritis/tenosynovitis are represented by difficulties in movement and lameness due to swelling of the limb joints and chronic inflammation of the synovial sheaths that generate aplomb deformities. The birds are lethargic, prefer support on the hocks, and crowd in feeding areas [5,14,20]. In the case of enteric infections (maldigestion/malabsorption syndrome), affected birds show growth retardation, persistent diarrhea, and ruffled and discolored plumage due to poor feed conversion. In the case of turkeys, nervous signs have also been reported; birds rest on their hocks and show varying degrees of torticollis [7,52].

It is important to distinguish between field infections, which frequently occur in association with other infectious agents and exhibit considerable variability in clinical signs and lesions, and controlled experimental infections, which allow for a clear demonstration of causality and the specific pathogenic mechanisms of avian reovirus.

## 6. Morphopathological Aspect and Intestinal Morphometry

Microscopically, in cases of malabsorption, at the intestinal level, atrophy of intestinal villi is observed, accompanied by varying degrees of crypt hypertrophy, inflammatory cellular infiltrate, and de-epithelialization. The lamina propria of the intestine contains not only mixed cellular infiltrate (macrophages, lymphocytes, and heterophils) but also intracytoplasmic eosinophilic inclusions, cytoplasmic vacuolization, and enterocyte necrosis [53,54]. Vacuolar degeneration of the epithelium and desquamation of the tips of the villi have been observed predominantly in the jejunum and ileum [55].

In the case of arthritis/tenosynovitis syndrome, macroscopic lesions in the limbs are limited to inflammatory and purulent edema around the tendons, thickening of the tarsal and femoral/tibial joints, which contain blood/purulent fluid. The distal extremity of the tibia may present erosions, extending to the bone plane, as well as stretching/rupture of the gastrocnemius tendon. Microscopically, at the articular level, hyperplasia of the synovial membrane is demonstrated by its infiltration with macrophages, lymphocytes, plasma cells, and numerous lymphoid aggregates. This cellular infiltrate can become exudative or fibrinous if present in the synovial cavity. The periarticular subcutaneous tissue not only demonstrates the same changes in terms of cellular infiltration (lymphocytes, plasma cells, and heterophils) but also hemorrhages or fibrin deposits [56]. The muscles demonstrate the phenomena of necrosis and resorption of muscle fibers accompanied by cellular infiltrate rich in macrophages, plasma cells, lymphocytes, and rare heterophils. The same pattern of inflammatory cellular infiltrate has been recorded at the tendon level [30,54,56,57].

### 6.1. Microscopic Lesions in Other Organs

In turkeys with nervous signs, microscopically, hypercellularity and perivascular clusters of lymphocytes and plasma cells have been observed in the nervous structure [51]. At the hepatic level, multifocal necrotic hepatitis has been noted, with varying degrees of severity, infiltration of multinucleated cells, and vacuolization of hepatocytes [22,54]. At the splenic level, focal necrosis with macrophage infiltration and fibrin deposits has been observed. At the cardiac level, infiltration of lymphocytes has been observed, along with macrophages and plasma cells (subepicardial myocarditis). In the kidneys, tubular degeneration and necrosis have been observed, along with perivascular inflammatory cellular infiltrate. Lymphocyte depletion of the follicles of the bursa of Fabricius has also been noted [54].

### 6.2. Histopathological Examination

Tissue fragments are fixed and embedded in paraffin according to the classical technique and then stained with hematoxylin/eosin (H&E). Histopathological preparations are made from each intestinal segment, and the preparations with the most obvious lesions from each segment are analyzed, taking into account the changes in the intestinal crypts. The intestinal crypts can be categorized as follows: normal in appearance, hypertrophied, or necrotic. Depending on the percentage of affected crypts, the intestines of the birds can be divided into three categories: (a) marked (over 30% of the crypts suffer changes); (b) moderate (15–30% of the crypts show changes); or (c) mild (under 15% of the crypts show changes). In addition, the cellular infiltrate must be classified according to the type of cells, their density, and their distribution in the tissues. Inflammatory cells are represented by plasmacytic macrophages, heterophils, and lymphocytes [58]. Morphometry is based on the evaluation of villus height and intestinal crypt depth, enabling a comparison between healthy birds and those with clinical signs, thus quantifying the degree of villus atrophy in the different intestinal segments. It is necessary to measure a minimum of 25 villi/crypts for each intestinal segment studied. Statistical analysis is performed with an analysis of variance test (Fisher test) using the STATA software version 15 (StataCorp, 2017, Stata Statistical Software: Release 15) [58].

## 7. Diagnosis

The presumptive diagnosis is established based on the epidemiological situation, clinical signs, and lesional aspects and is confirmed by laboratory tests. Confirmation of this disease involves the isolation and identification of the virus. It can be achieved in the following ways: (a) cultivation on cell lines; (b) detection of viral RNA and amplification of specific genes (σC gene) via PCR or RT-PCR (reverse transcription polymerase chain reaction) techniques; (c) genomic sequencing (NGS), which allows for the identification of viral diversity at the intestinal level or in tissues; (d) ELISA (Enzyme-Linked Immunoassay), for the detection of antibodies in serum; or (e) histological examination supplemented with immunohistochemical examination (IHC), which allows for the identification of viral antigen in tissues [4,5,26,41,59,60,61,62,63].

The virus can be isolated by cultivation on embryonated eggs. PCR-positive reovirus samples will be inoculated into the yolk sac of SPF embryos (without specific pathogens), thus achieving viral multiplication. This is followed by a passage on liver cell cultures of SPF embryos, recording the specific cytopathic effect [4]. When the viral material is inoculated into the yolk sac, embryo death occurs in the first 5 days, and when the viral material is placed on the chorioallantoic membrane, embryo death occurs after 7 days. Multiple necroses of the spleen, liver, and chorioallantoic membrane are among the lesions of infected embryos [30].

In experiments, some researchers managed to inoculate a virulent strain in chicks that were only 2 days old. They were infected through oral and intratracheal routes, and the reovirus was identified in the organs collected within the first 9 days of inoculation by RT-PCR [5,7,41,64]. Samples collected for analysis may come not only from broilers, breeding birds, or laying hens with clinical signs of malabsorption syndrome but also from healthy ones in order to identify and characterize new pathogens involved in this pathology. Samples of intestinal contents, various organs, or blood can be analyzed. The collection and preparation of samples for the identification of pathogens is performed differently depending on the nature of the material chosen for analysis and the technique used [64]. The histological examination is performed classically. After embedding the tissue fragments in paraffin and sectioning, they are fixed, clarified, and stained with hematoxylin/eosin (H&E). The histopathological examination is completed with an immunohistochemical examination (IHC). After deparaffinization, the samples are rehydrated, and finally, the antigens are recovered. They are incubated with primary and secondary antibodies, counterstained with hematoxylin, and evaluated microscopically using positive and negative controls. During microscopic evaluation via immunohistochemistry, positive signals are observed in the form of brown staining in the cytoplasm of epithelial cells in the small intestine (jejunum/ileum), indicating the presence of reovirus viral antigens in these cells [64].

The differential diagnosis of arthritis/tenosynovitis syndrome is made against bacterial arthritis, caused by infection with *Mycoplasma synoviae*, *Streptococcus* spp., or *Staphylococcus* spp., which can cause swelling of the tibiotarso-metatarsal joint and aplomb defects [32]. The most frequently isolated species of staphylococci are represented by *S. xylosus*, *S. arlettae*, *S. cohnii*, and (to a limited extent) *S. aureus* [48].

**Figure 1:**
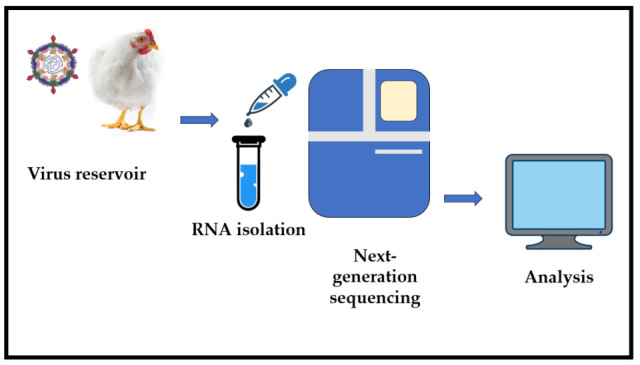
Viral identification by NGS.

Next-generation sequencing (NGS) allows for comparative metagenomic analysis of gut contents from healthy/clinical birds, without the need for virus cultivation or prior knowledge of the target sequence, making it ideal for metagenomic studies and the identification of new and emerging pathogens. The main steps of the NGS technique involve sample collection and processing, viral DNA/RNA extraction, genome amplification, genomic library construction, actual sequencing, and bioinformatic analysis (Figure 1) [41,60].

From the intestinal samples of birds with clinical signs, a few grams of content from the intestinal lumen are removed; then, in a sterile centrifuge tube, the following are added: phosphate-buffered saline and the rest of the intestinal content. After homogenization and centrifugation, the supernatant is removed and then ultracentrifuged, and the sediment is reconstituted with a small amount of phosphate-buffered saline in order to eliminate exogenous nucleic acids [41]. To eliminate contaminating genetic material, the viral suspension is incubated at 37 °C for 30 min, followed by inactivation using the DNase 1 kit and incubation at 65 °C for 10 min. This step ensures the elimination of free nucleic acids. Subsequently, the samples are placed on ice and divided for DNA and RNA extraction. Viral RNA and DNA are extracted separately using commercial kits (Ribopure RNA, Life Technologies, Carlsbad, CA, USA and Viral RNA Mini Kit, Qiagen, Manchester, UK), according to the manufacturer’s instructions, ensuring the procurement of quality nucleic acids for subsequent steps. To obtain sufficient quantities of genetic material, whole-genome amplification (WGA) and transcriptome amplification (WTA) reactions are applied using the commercial kits Repli-g Cell WGA and WTA Kit (Qiagen^®^, Manchester, UK), according to the manufacturer’s instructions [41]. The resulting genomic material is subjected to high-frequency sound waves (sonication) to obtain fragments of optimal sizes required for the sequencing platforms used (GS Junior, FLX+, and MiSeq). After sonication, the purified samples are subjected to fragment repair, adapter ligation, and removal of small fragments. The quality of the libraries is assessed using the 2100 Bioanalyzer and quantified fluorometrically, with subsequent performance of emPCR (Emulsion PCR). The actual sequencing is performed using the three platforms GS Junior, FLX+, and MiSeq. Samples are sequenced using 454 and Illumina MiSeq platforms. The resulting reads are assembled into contigs and analyzed using BLAST and MEGAN (version 4) [41] for taxonomic identification [41]. Over 2 million reads can be generated, which are assembled into contigs that are assigned to distinct viral families/genera, highlighting the diversity of the enteric virome [41].

Intestinal content samples will undergo processes of homogenization, centrifugation, filtration, and precipitation of viral particles, followed by extraction of viral nucleic acids using the ZR and ZR-96 viral DNA/RNA kits. Complementary DNA synthesis is performed, and the DNA library is prepared using the Nextera XT (Illumina, San Diego, CA, USA) kit. Deep sequencing is performed on the Illumina MiSeq platform, using the Nextera™ XT sample preparation kit [17]. The obtained data are assembled and analyzed bioinformatically. Viruses are identified by sequence alignment using BLASTx and BLASTn, and viral genomes are mapped using the Geneious R9 software (Biomatters Ltd. L2, 18 Shortland Street Auckland, 1010, New Zealand) [60]. The phylogenetic analysis focuses on the NS and VP1 genes, performing sequence alignment with the online service MAFFT, and finally creating an identity map produced by the Geneious R9 software. To identify possible recombination events, the complete genomes are analyzed with the GARD algorithm [60]. Finally, structural modeling of the viral capsid proteins VP1 and VP2 is performed using AlphaFold3 and PyMOL, and the location of the B and T epitopes is estimated using NetMHCpan and NetMHCIIpan [60]. Structural modeling of the capsid proteins allows for the comparison of identified viruses, and the localization of the epitopes allows for the determination of potential immunogenic regions of the viruses [60].

Experimentally, the infection can be reproduced by culturing the virus on embryos from SPF eggs (pathogen-free) to evaluate the clinical progress and their pathogenicity in broiler chickens. Subsequently, a viral dose is established and inoculated either through the oculonasal route or oral administration (directly into the goiter). Complete sequencing of the viral genome is performed by extracting RNA from the intestine of infected embryos, using the TRIzol reagent. After removing DNA and rRNA, cDNA libraries are constructed and subsequently sequenced using Illumina technology. For the assembly and validation of the sequences, the Geneious Prime and SPAdes software (version 2020.1.1) are used, resulting in a nucleotide identity of 100%. Phylogenetic analysis is performed using MAFFT [61].

To detect the viral load, post-infection lacrimal and cloacal swabs are collected and analyzed by RT-qPCR. Several birds are euthanized, and intestinal and tracheal samples are subjected to histopathological and IHC examination [61].

Serums collected from the birds are tested by ELISA, and the titers obtained will indicate the presence or absence of maternal antibodies. For histological and IHC analysis, tissues are processed and stained with hematoxylin/eosin (H&E), according to the classical method, and the presence of viral antigen is detected by immunohistochemistry, using anti-matrix and anti-spike monoclonal antibodies. The data obtained regarding viral load, clinical scores, and batch uniformity are statistically analyzed [61].

Controlled experimental studies offer superior evidentiary value by enabling the direct demonstration of a causal link between orthoreovirus infection and clinico-pathological outcomes in broiler chickens while minimizing confounding factors typical of field studies.

### 7.1. Confirmation of Reovirus Infection by RT-PCR

Organ samples (small intestine, proventriculus, tendon, yolk sac, kidney, trachea, and liver) are processed via homogenization, centrifugation, and filtration. Cells are cultured in DMEM medium with fetal bovine serum and antibiotics, which are incubated at 37 °C. The specific cytopathogenic effect of reovirus appears after 5–6 days and is marked by the formation of giant cells and syncytia [65].

Viral RNA is extracted from cell suspensions, and subsequently, the amplification of the reovirus σC gene is performed using the RT-PCR technique, with specific primers. The PCR product is sequenced by the Sanger method. The sequences obtained are deposited in a database (GenBank) [65]. Data analysis allows for the identification and comparison of the genetic sequences obtained, establishing the phylogenetic relationships between the isolates. The use of bioinformatics tools such as BLAST, MEGAX, and Geneious confirms gene homology and highlights the diversity and evolution of reoviruses [60].

### 7.2. Detection of Antibodies Specific to Avian Orthoreovirus by ELISA Method

Blood samples are collected by puncturing the wing vein. Serum is obtained after coagulation and centrifugation, heat inactivation, and storage at −20 °C. After blood collection, the birds are euthanized, and the proventriculus and pancreas are collected aseptically. The tissues are homogenized in phosphate-buffered saline (PBS) to obtain a suspension. This is clarified by centrifugation and stored at −80 °C until use [59].

Avian orthoreovirus (ARV)-specific antibodies are detected by testing the collected sera with the ELISA method, using a commercial kit. Sera are diluted 1:500 and deposited on ELISA microplates coated with ARV antigen. After processing, the results are expressed as a sample/positive ratio (S/P), and titers are calculated. Samples that record ratios (S/P) and titers above a certain standard value (S/P > 0.2) are considered positive for avian orthoreovirus (ARV) antibodies [59].

## 8. Prevention and Control

Orthoreovirus infections represent a significant threat to the global poultry industry, causing significant economic losses through reduced production performance, mortality, and bird welfare. Despite vaccination of poultry flocks with classical commercial strains, such as S1133, 1733, 2177, 2035, 2408, and SS412, multiple outbreaks of the disease have been reported (Table 1) [49,66].

Phylogenetic analysis of the sigma C gene of avian orthoreoviruses isolated from diseased birds classifies them into seven phylogenetic clusters, six of which are labeled I to VI, with a distinct cluster labeled Eu (a cluster different from those identified in Europe). This genotypic divergence indicates that the circulating strains belong to novel antigenic lineages, which are not immunologically covered by the current vaccine (Table 1) [5,49,67,68,69,70,71].

Therefore, the correlation between genotype (variability of the sigma C gene sequence), phylogenetic cluster, and lack of relatedness to the vaccine strain suggests a weakening of immune protection in flocks of birds immunized with commercial vaccines (Table 1) [5,49,67,68,69,70,71].

These data come from field investigations that accurately reflect the epidemiological situation in commercial farms; however, their evidentiary value is limited, as technological factors, nutrition, and coinfections can influence the clinical presentation and disease progression.

Vaccination with the S1133 strain is commonly used in breeder flocks; however, in the case of broiler chickens, the use of this strain poses significant risks. Neonatal vaccination with the S1133 strain disrupts the functionality of the gastrointestinal tract in chicks, leading to poor feed conversion and reduced weight gain. Moreover, the cost/benefit ratio does not justify the implementation of this prophylactic measure from a farm management and economic standpoint [50]. Furthermore, maternally derived antibodies transmitted to chicks from breeder hens vaccinated with the commercial S1133 strain have failed to prevent infection with the live-modified S1133 strain in broilers. Given that viral replication occurs in the gastrointestinal tract regardless of the presence or absence of maternal antibodies, neonatal vaccination of broilers is not justified [50].

The circulation of emerging viral strains that are genetically distinct and do not overlap with vaccine strains, as well as the resistance of viruses to environmental factors, makes it difficult to control this disease. Non-specific disease prevention measures require compliance with breeding technology, disinfection, and rigorous hygiene. It is preferable to use only eggs from breeding birds from disease-free flocks for incubation. Over time, live and inactivated vaccines have provided protection against avian reovirus, with the major objective being the prevention of vertical transmission. Currently, vaccines on the market do not protect against the multitude of orthoreovirus strain variants, which is why the development of new vaccines is necessary [20,72]. Genetic recombinations, the diversity of the σC gene (main determinant of virulence and antigenic variation), and the high mutation rate influence the efficiency of vaccination, making some routine immunizations ineffective. Under these conditions, autogenous (locally adapted) vaccines become a promising option for the effective control of avian reovirus, especially in farms where autochthonous genetic variants are present [66,72].

In addition to conventional and autogenous vaccines, recent studies have focused on the development of innovative technologies. Lipid nanoparticle (LNP)-encapsulated mRNA vaccines have demonstrated protective efficacy in broilers against divergent H5 avian influenza strains, eliciting both humoral and cellular immune responses while maintaining a reduced reactogenicity profile [73,74]. At the same time, virus-like particles (VLPs), including chimeric constructs or those generated through baculovirus-based systems or plant expression platforms, have shown high levels of immunogenicity and contributed to limiting the replication of respiratory and enteric viruses, such as infectious bronchitis virus and avian influenza virus [75,76,77]. With regard to avian orthoreoviruses (ARVs), proof-of-concept studies have demonstrated that the σC protein can be expressed as a recombinant antigen (in plants or via bacterial vectors), inducing specific immune responses [78,79]. Although these platforms are still at an experimental stage and not yet commercially available, they represent promising directions for the development of next-generation ARV vaccines.

Specific maternal anti-reovirus antibodies, passively transferred from the breeder hen to the chick through the yolk sac, provide temporary protection during the first days of life, reducing the risk of early clinical infection and the severity of joint and intestinal lesions [40]. However, high maternal antibody titers can interfere with the replication of live vaccine viruses, thereby affecting the induction of active immunity following vaccination. Consequently, the optimal timing of chick vaccination should be determined based on the maternal antibody titer in order to avoid interference and ensure an effective immune response [40].

Thus, the recommended prophylactic and control measures include continuous monitoring, either through serological testing or molecular sequencing; the periodic updating of commercial vaccines; and the development of autogenous vaccines, as well as next-generation platforms such as lipid nanoparticle-encapsulated mRNA vaccines and chimeric vaccines. These strategies must be complemented by strict biosecurity measures and further studies addressing pathogenicity and immunogenicity [49,66,72].

## 9. Conclusions

Avian orthoreovirus infections continue to represent a major challenge for the poultry industry, especially in the context of intensive broiler chicken farming. This article highlights the fact that the fast but incomplete development of the post-hatch digestive tract, together with immunological immaturity, favors the occurrence of malabsorption syndrome, a multifactorial condition where the association of enteric viruses plays an essential role. ARV induces immunosuppression and alters intestinal morphology, thereby impairing nutrient absorption. The diversity of viral genotypes, as well as the lack of exact correlations between genotype and clinical signs, complicates the current immunization schedule.

Modern diagnostic methods, particularly next-generation sequencing (NGS), enable precise characterization of the intestinal virome and the identification of emerging viral strains. However, the limited efficacy of current commercial vaccines highlights the need for the development and investigation of innovative approaches in poultry production. Autogenous vaccines tailored to local viral strains, mRNA vaccines encapsulated in lipid nanoparticles (LNPs), chimeric vaccines, and baculovirus-based vaccines have shown significant potential in avian medicine by inducing high levels of immunogenicity, thus representing promising future directions for the immunization of poultry against avian reovirus.

Additional studies are needed regarding the pathogenesis of enteric virus infections and viral replication in the intestines of broilers. The corroboration of qualitative and quantitative virological examinations, as well as the quantification of morphopathological lesions in the intestine, could truly establish whether avian orthoreovirus is the determining etiological agent of malabsorption syndrome in broilers or if malabsorption is the result of mixed viral infections.

## Figures and Tables

**Table 1:** Distribution of avian orthoreovirus (ARV) viral strains.

Continent	Country	Vaccine Strains	Isolated Strains	Genotypic Clusters (σC)	References
Africa	Egypt	S1133, 2177, 1733, 2408, SS412,	D257, D2248	I, II, III, IV, V	[36,47]
	Tunisia	S1133	TU430, TU105B6, TU5, TU97.2	Eu	[47]
North America	Pennsylvania	S1133, 1733, 2048	Reo/PA/Broiler/05273a/14 Reo/PA/Broiler/07634/14 Reo/PA/Broiler/30857/11 Reo/PA/Broiler/07209a/13 Reo/PA/Broiler/03476/12, etc.	I, II, III, IV, V, VI	[32,47]
	Canada	S1133, 1733, 2048	14-0041-Broiler-SK-2014 16-0711-Broiler-BC-2016 12-1009-Broiler-AB-2012 15-0157-Broiler-BC-2015 17-0025-Broiler-AB-2017, etc.	I, II, III, IV, V, VI	[47]
	California	S1133, 1733, 2048	MK247050, MK246988, MK247008, MK247040, MK247049, etc.	I, II, III, IV, V, VI	[47]
Asia	South Korea	av-S1133, S1133	SD09-1, LN09-1, GX110116, K1600657	I, Eu	[25,47]
	Israel	S1133	ISR-59103, ISR-5216	I, II, III, IV,	[17]
	Iran	S1133	ARV1IR019, ARV2IR018	II, IV	[47]
	Taiwan	S1133	GA/12355, GA/12274, 1017-1, T6, 916, etc.	I, II, III, IV, Eu	[4,47]
	China	S1133	LN160607-1, GX150816, SD150806, JS170705-1, HeN130728, etc.	II, III, VI, Eu	[47]
	Japan	S1133	ARV Bro NGN20 7-1 b, ARV Bro GF20 4-1 a, JP/Tottori/201, JP/Nagasaki/2017, OS161, etc.	II, IV, V, VI, Eu	[47]
Europa	Germany	S1133	GEI10 97M, GEL01 96T, GEL03 97T, GEL13 98M, GEL01 96T, etc.	I, II, III, IV, V, Eu	[4,47]
	France	S1133	11-17268, 12-1167, 11-12523.	Eu	[47]
	Hungary	S1133, 1733, 2408	HUN392, HUN290, HUN131, HUN-142, HUN-143, etc.	II, IV II, III, IV, V, Eu	[16,47]
	Romania	S1133	ROM11, ROM6, ROM8	II, IV II, III, IV, V, Eu	[16,47]
	Ukraine	S1133	UKR1	IV	[47]
	Russia	S1133	RUS1	IV	[47]
	Netherlands	S1133	NLA13 96T, NLI12 96M, NLI02 98 M, NLI20 98M, NLI03 92T, etc.	I, II, III, IV, Eu	[4,47]
South America	Brazil	S1133 and 2177	USP_BR_336-15, USP_BR_358-9, BR-5881, BR/2290, BR/3292, etc.	I, II, VII	[13,47]
Oceania	Australia	S1133, 2408, 1733	SOM-4, RAM-1, SOM-4, RAM-1	I, II, III, IV, V, VI	[4,45,47]

## Data Availability

The original contributions presented in this study are included in the article. Further inquiries can be directed to the corresponding authors.
